# Antibodies to the Novel Human Pegivirus 2 Are Associated with Active and Resolved Infections

**DOI:** 10.1128/JCM.00515-16

**Published:** 2016-07-25

**Authors:** Kelly E. Coller, Michael G. Berg, Matthew Frankel, Kenn Forberg, Rita Surani, Charles Y. Chiu, John Hackett, George J. Dawson

**Affiliations:** a Abbott Laboratories, Abbott Park, Illinois, USA; b Department of Laboratory Medicine, University of California, San Francisco, California, USA; c UCSF-Abbott Viral Diagnostics and Discovery Center, San Francisco, California, USA; d Department of Medicine, Division of Infectious Diseases, University of California, San Francisco, California, USA; Memorial Sloan-Kettering Cancer Center

## Abstract

A novel blood-borne human pegivirus (HPgV), HPgV-2, was recently identified in hepatitis C virus (HCV)-infected individuals and individuals who had received multiple transfusions. Robust serological assays capable of detecting antibodies in HPgV-2-infected individuals are needed to establish global seroprevalence rates and potential disease associations. The two objectives of this study were to determine the utility of mammalian cell-expressed HPgV-2 E2 glycoprotein or bacterium-expressed nonstructural protein 4AB (NS4AB) in detecting past or present infections and to compare the total prevalence (antibody and RNA positive) of HPgV-2 with that of the other human pegivirus, HPgV-1 (GB virus C [GBV-C]). HPgV-2 E2 antibodies were detected in 13 (92.86%) of 14 HPgV-2-viremic cases, and NS4AB antibodies were detected in 8 (57.14%) of 14 cases. The HPgV-2 seroprevalence was significantly higher (*P* < 0.0001) among HCV-infected individuals (3.31% [24 of 726 samples]) than among non-HCV-infected individuals (0.30% [4 of 1,348 samples]). Of 31 anti-E2-positive samples, 22 had supplemental supporting data; 12 samples were HPgV-2 RNA positive and 10 nonviremic samples were antibody positive for peptides or NS4AB. The total prevalence of HPgV-1 (35.00%) was significantly higher than that of HPgV-2 (1.33%) in all populations tested (*P* < 0.0001). For HPgV-1, codetection of antibodies to E2 and RNA was infrequent (5.88%). In contrast, antibodies to E2 were detected in most HPgV-2-viremic individuals (92.86%), as is observed among individuals chronically infected with HCV, most of whom are antibody positive for HCV E2. Our studies indicate that HPgV-2 circulates with HCV and displays a profile similar to the serological profile of HCV-infected persons, although the pathogenicity of this virus has yet to be established.

## INTRODUCTION

Two recent independent reports describe the discovery of a novel human pegivirus (HPgV) of the family Flaviviridae, which has been provisionally designated human hepegivirus 1 (HHpgV-1) (1) or human pegivirus 2 (HPgV-2) (2). HPgV-2 and HHpgV-1 are different strains of the same virus (96% identity) and are distinct from the only other known human pegivirus, HPgV-1 (GB virus C [GBV-C]). For this report, the HPgV-2 designation is used. Both studies indicated that HPgV-2 is a highly divergent virus, is blood borne, and exhibits a low prevalence of viremia in populations at risk for parenteral exposure. In the study by Kapoor et al., 4 (1.82%) of 220 samples in cohorts with transfusion transmission or hemophilia were found to be viremic; 3 of those 4 samples were also viremic for hepatitis C virus (HCV) (1). In the study by Berg et al., 11 (0.45%) of 2,440 samples were viremic for HPgV-2, with all cases being found among individuals with active HCV infections, despite screening of other high-risk groups (HIV and hepatitis B virus [HBV] groups) and volunteer donors (2). Given that approximately 75% of HCV infections are associated with intravenous drug use (3), these data suggest that HPgV-2 is likely transmitted by a parenteral route. Similar to findings for HCV, it was noted that HPgV-2 viremia persisted for up to 5.4 years, indicating an ability to establish chronic infections (1).

The first serological assay for detection of HPgV-2 antibodies used peptides designed for regions selected from the index case sequence (2). Three peptides from nonstructural proteins (NSs) were found to be useful for identification, identifying 9 (75.00%) of 12 HPgV-2 RNA-positive samples. An antibody test using peptide 16 (P16), encompassing overlapping regions of NS4A and NS4B, had the highest rate of detection, identifying 8 of 12 HPgV-2 RNA-positive samples and resulting in a positive predictive value of 66.70%. The design of P16 was based on the HCV 5-1-1 epitope within the C100-3 antigen of HCV, which also encompasses the NS4A/B region and is an immunodominant marker for HCV infection (reviewed in references 4 and 5). While no single marker emerged with a 100% predictive value for viremia, the data presented are similar to those for HCV, in that single peptides (core peptide, NS4, and NS5A) can be useful as markers for the identification of HCV-infected individuals (6). In contrast, the use of peptides or prokaryotically expressed antigens for nonstructural proteins had limited utility as serological markers for HPgV-1 (GBV-C) (7). In order to expand on the first serological study, a recombinant antigen was designed for the NS4AB region that included the P16 peptide sequence.

In the family Flaviviridae, the envelope proteins are the major structural components of the virion surface and are important targets of the humoral immune response (8, 9). For many flaviviruses that do not establish chronic infections, such as West Nile virus, dengue virus, and yellow fever virus, the appearance of antibodies to envelope proteins is an indicator of resolving or resolved infections (8, 10–13). Notably, for HPgV-1, antibodies to mammalian cell-expressed envelope glycoprotein E2 are associated with resolving or resolved infections, and individuals who do not develop detectable antibodies to the envelope protein establish chronic infections (14–17). In contrast, during HCV infection, chronically infected individuals maintain active infections in the presence of envelope protein-specific antibodies (18–21). Mammalian expression constructs of HPgV-1 and HPgV-2 E2 were designed, to determine whether the appearance of antibodies to E2 served as a marker for resolving/resolved infections or active infections.

One of the key first steps in establishing the prevalence and potential pathogenicity of HPgV-2 is to develop serological and molecular tests that enable accurate identification of individuals who have been infected. In the current study, we designed improved serological assays for HPgV-2, using recombinant viral proteins, and we used them to compare the seroprevalence of HPgV-2 with that of the other human pegivirus, HPgV-1 (GBV-C).

## MATERIALS AND METHODS

### Expression and purification of HPgV-2 NS4AB.

The Protean 3D program (DNASTAR, Madison, WI, USA) was used to predict that an 81-amino acid segment of HPgV-2 NS4A/4B (derived from GenBank accession number KT427414.1) would be localized to the cytoplasm. This region was chosen for expression in the pMAL-C5X vector, which, under the control of an isopropyl-β-d-thiogalactopyranoside (IPTG)-inducible promoter, allowed for the addition of a maltose-binding protein (MBP) tag at the amino terminus and a 6-histidine tag at the carboxyl terminus (Genscript, Piscataway, NJ, USA). The construct was expressed in Escherichia coli strain BL21(DE3). Following IPTG induction for 4 h at 37°C, cells were lysed, and soluble protein was purified using the ProBond purification system (Invitrogen, Grand Island, NY, USA). Western blotting of the purified protein was performed using a WesternBreeze chromogenic kit (Invitrogen), and purified protein was detected using an anti-His antibody (Invitrogen). Protein was visualized using 5-bromo-4-chloro-3-indolylphosphate (BCIP)/nitroblue tetrazolium (NBT) staining (Novex by Life Technologies) and a Bio-Rad Gel Doc EZ imager, using Image Lab v4.0 software.

### Expression and purification of E2 glycoproteins from HPgV-1 and HPgV-2.

Two separate expression constructs were designed to express the HPgV-1 E2 glycoprotein and the HPgV-2 E2 glycoprotein (GenBank accession number KT427414.1). The predicted ectodomain of each glycoprotein was subcloned into a mammalian expression vector containing a cytomegalovirus (CMV) promoter and a signal sequence encoding a leader peptide. An 8-histidine tag was cloned in frame at the carboxyl terminus of each E2 open reading frame (ORF), for purification. Cultures of HEK293-6E cells were transiently transfected with each plasmid individually, using polyethylenimine (PEI). Cells and supernatants were collected 4 days posttransfection and centrifuged for 10 min at 2,000 rpm, and the supernatants were concentrated using the Millipore Cogent μScale system. HPgV-2 and HPgV-1 E2 proteins were purified from the concentrated supernatants using the ProBond nickel purification system (Invitrogen). Cell lysates, concentrated supernatants, and purified proteins were run on a 4% to 20% SDS-PAGE gradient gel (Novex; Life Technologies), and Western blotting was performed using a WesternBreeze kit (Invitrogen) with an alkaline phosphatase-conjugated anti-His primary antibody (Novex; Life Technologies).

### Immunofluorescence.

To determine the intracellular localization of the HPgV-2 and HPgV-1 E2 constructs, immunofluorescence analysis was performed with COS-7 cells that had been transiently transfected, using Lipofectamine 2000 (Invitrogen), with HPgV-2 E2 DNA, HPgV-1 E2 DNA, or no DNA (negative control), according to the manufacturer's instructions. Cells were plated onto poly-l-lysine-treated coverslips 1 day posttransfection, followed by fixation using 4% paraformaldehyde. Coverslips were washed three times with phosphate-buffered saline (PBS), followed by blocking for 1 h at room temperature in PBS with 5% bovine serum albumin (BSA) and 0.3% Triton X-100, with rocking. Both primary (anti-His) and secondary (Alexa Fluor 488) antibody incubations were carried out in PBS with 1% BSA and 0.3% Triton X-100. Coverslips were mounted onto slides using ProLong Gold reagent plus 4′,6-diamidino-2-phenylindole (DAPI) (Thermo Fisher). Images were obtained using Metamorph software and a 20× objective on a Nikon (TE2000) inverted microscope, with fluorescein isothiocyanate (FITC) and DAPI filter cubes.

### Detection of HPgV-2 antibodies by slot blotting.

Purified human IgG (Southern Biosciences), NS4AB, HPgV-2 E2, and HPgV-1 E2 were diluted in 50 mM 3-morpholino-2-hydroxypropanesulfonic acid (MOPSO) buffer (pH 7.0) at concentrations of 0.5 mg/ml (IgG), 10 and 100 μg/ml (NS4AB), and 10 and 100 μg/ml (HPgV-1 E2 and HPgV-2 E2) and were applied to individual channels of the slot blot apparatus (Immunetics Miniblotter 28SL) containing a nitrocellulose membrane. Protein was allowed to adhere for 1 h at room temperature, with rocking, followed by aspiration of unbound protein. Membranes were washed twice with TNT wash buffer (20 mM Tris-HCl, 0.5 M NaCl, 0.3% Tween 20 [pH 8.0]) and twice with 1× PBS and then were blocked for 30 min at room temperature in 1× PBS with 5% nonfat dry milk, with rocking. Membranes were washed twice with 1× PBS, after which they were cut into strips.

For immunodetection, each strip was incubated with sample diluent (TNT wash buffer with 5% nonfat dry milk and 10% heat-inactivated calf serum) for 10 min at room temperature, with rocking. Fifteen microliters of sample was added to each well containing a strip and the samples were incubated for 2 h at room temperature, followed by aspiration and three washes with TNT wash buffer. Strips were incubated for 1 h with secondary antibody [alkaline phosphatase-conjugated goat anti-human IgG(H+L); Southern Biotechnologies] in sample diluent, followed by aspiration and two washes with TNT wash buffer. Bound antibodies were detected using SigmaFast BCIP/NBT tablets dissolved in distilled water. Strips were allowed to develop for 15 min and then washed three times with distilled water.

### Sample collection.

Samples evaluated by Berg et al. (2) were retested if adequate volumes were available. Overall, the following samples were purchased for HPgV-2 and HPgV-1 prevalence studies: 456 HBV-positive samples (300 from the American Red Cross [Gaithersburg, MD, USA] and 156 from ProMedDx [Norton, MA]), 434 HIV-positive samples (200 from the American Red Cross and 234 from ProMedDx), 726 HCV RNA-positive/antibody-positive samples (440 from the American Red Cross and 286 from ProMedDx), and 299 HCV RNA-negative/antibody-positive samples from the American Red Cross. A volunteer donor population consisting of 416 volunteer plasmapheresis donors from the southern Midwest region of the United States (Golf Coast Regional Blood Center, Houston, TX) was used to establish a cutoff value. The negative control consisted of pooled plasma samples negative for HCV, HIV, HBV, HPgV-1 RNA, and HPgV-1 antibodies (Architect HPgV-1 E2 assay).

### Molecular screening.

A multiplex quantitative PCR (qPCR) assay using primers and probes directed against the HPgV-2 targets (5′ untranslated region [UTR] and NS2/3) and the HPgV-1 target (5′ UTR) was used to screen the samples described above for the presence of HPgV-2 and HPgV-1 viral RNAs (2).

### Architect testing.

Magnetic microparticles (Spherotech, Lake Forest, IL, USA) were coated with 50 μg of NS4AB or 100 μg of purified HPgV-2 or HPgV-1 E2, using *N*-(3-dimethylaminopropyl)-*N*′-ethylcarbodiimide hydrochloride (EDAC) (Sigma-Aldrich, St. Louis, MO, USA) to cross-link the protein to the microparticles. Cross-linking was performed for 3 h at room temperature with end-over-end mixing, after which unbound protein was washed away. Coatings were performed with 1.0% solids, and the mixtures were diluted to 0.05% solids for testing.

An indirect immunoassay to detect IgG antibodies to HPgV-2 NS4AB, HPgV-2 E2 glycoprotein, or HPgV-1 E2 glycoprotein was adapted to the Architect immunoanalyzer (Abbott Laboratories). Briefly, sample, diluent, and coated microparticles were incubated together for 18 min, followed by a wash step to remove unbound antibodies. Bound antibodies were detected following a 4-min incubation with an acridinium-anti-human IgG conjugate. Unbound conjugate was washed away, and signals were detected by chemiluminescence and quantified as relative light units (RLUs).

A volunteer donor population (*n* = 416) was used to establish a cutoff value. The volunteer donor samples were determined to be negative for HPgV-2 RNA, antibodies against HPgV-2 peptides, and antibodies against HPgV-2 recombinant proteins (NS4AB and E2). Since the seroprevalence is low among volunteer donors (2), the median result was predicted to be negative for antibodies to HPgV-2, with sample results similar to the median. As the cutoff value, we chose the sum of the median signal plus the signal representing 7 standard deviations (SDs) from the median for all populations tested, for antibodies to HPgV-1 E2, HPgV-2 NS4AB, and HPgV-2 E2. Infrequently occurring outlier samples with signals 10 times the median were not included in the calculation of the cutoff value.

### Statistical analysis.

The 95% confidence intervals (CIs) were calculated to determine prevalence differences between HPgV-2 and HPgV-1. Fisher's exact test was used to calculate all *P* values.

## RESULTS

### Expression and purification of HPgV-2 NS4AB.

**FIG 1:**
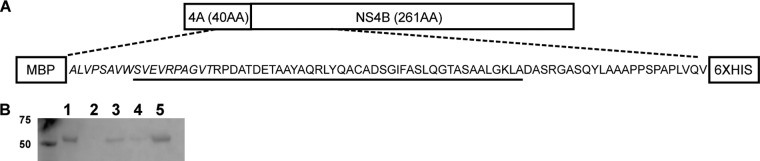
Expression and purification of the NS4AB recombinant protein. (A) Design of the NS4AB recombinant protein. The NS4A sequence is italicized, and the sequence representing peptide 16 (P16) is underlined. The expected size of the recombinant protein is 54 kDa. AA, amino acids. (B) Western blot of fractions from the Ni+ column purification of the NS4AB recombinant antigen, detected using an anti-His antibody. Lane 1, induced cytoplasmic lysate; lane 2, flowthrough fraction; lane 3, wash 1; lane 4, wash 2; lane 5, eluted protein.

An NS4AB recombinant protein construct encompassing the P16 region, which was shown previously to be a marker of HPgV-2 infection (2), and incorporating an additional NS4AB sequence (Fig. 1A and B) was expressed in E. coli and purified. Alignment of the HPgV-2 NS4AB region indicated low levels of amino acid identity with HCV (14.0%) and HPgV-1 (22.2%), thus minimizing the potential for antibody cross-reactivity (see Fig. S2A in the supplemental material).

### Expression and purification of HPgV-2 and HPgV-1 E2 recombinant proteins.

**FIG 2:**
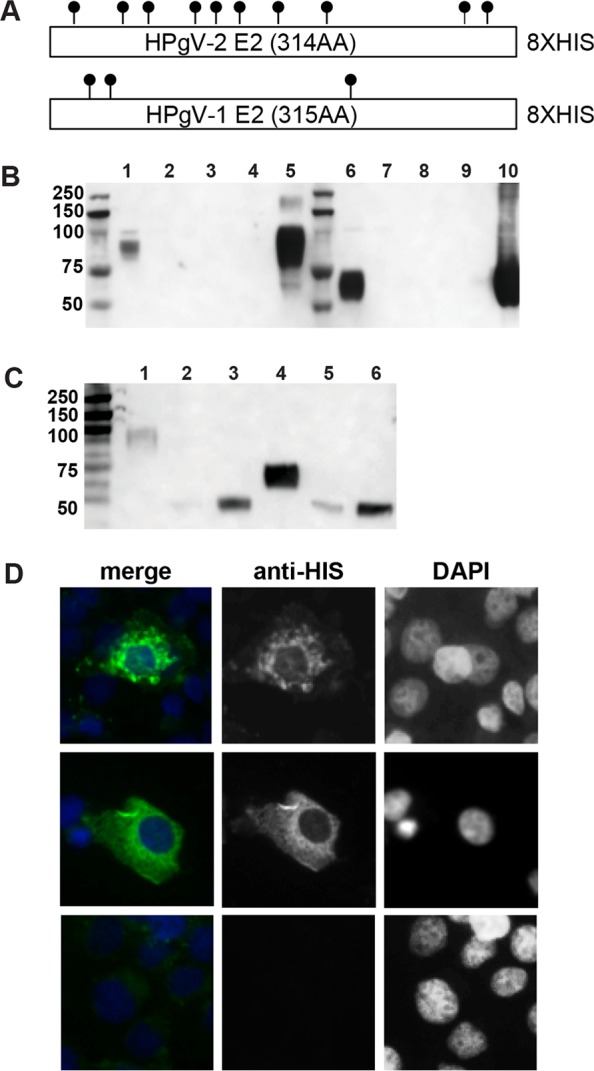
Design, purification, and characterization of HPgV-2 E2 and HPgV-1 E2. (A) Design of the E2 expression plasmids. The ectodomain of each E2 protein was expressed in a mammalian expression vector containing a leader sequence. For the 314-amino acid (AA) HPgV-2 E2, the expected size was 35.2 kDa; for the 315-amino acid HPgV-1 E2, the expected size was 33.6 kDa. Predicted *N*- and *O*-linked glycosylation sites are indicated. (B) Western blot of fractions from the Ni+ column purification of HPgV-2 E2 and HPgV-1 E2, using an anti-His antibody. Lanes 1 to 5, HPgV-2 E2; lane 1, input; lane 2, flowthrough fraction; lane 3, wash 1; lane 4, wash 2; lane 5, eluted protein; lanes 6 to 10, HPgV-1 E2; lane 6, input; lane 7, flowthrough fraction; lane 8, wash 1; lane 9, wash 2; lane 10, elute. (C) Western blot of samples, using an anti-His antibody, after PNGase F deglycosylation of purified proteins. One or 10 μg of purified HPgV-2 E2 or HPgV-1 E2 was treated with PNGase F. Lanes 1 to 3, HPgV-2 E2; lane 1, untreated protein; lane 2, 1 μg treated with PNGase F; lane 3, 10 μg treated with PNGase F; lanes 4 to 6, HPgV-1 E2; lane 4, untreated protein; lane 5, 1 μg treated with PNGase F; lane 6, 10 μg treated with PNGase F. (D) Immunofluorescence of COS-7 cells transfected with HPgV-2 E2 or HPgV-1 E2 expression constructs. (Top) HPgV-2 E2. (Middle) HPgV-1 E2. (Bottom) no-DNA control. Magnification for all panels, ×20.

Both HPgV-1 E2 and HPgV-2 E2 contain multiple predicted *N*- and *O*-linked glycosylation sites (Fig. 2A), and recombinant protein constructs were designed for mammalian expression, to facilitate secretion into the supernatant. Western blotting and anti-His detection indicated that proteins were indeed secreted into the supernatant fluid; however, both proteins had greater electrophoretic mobilities than predicted for molecular masses of 39.6 kDa (Fig. 2B, lanes 1, 5, 6, and 10). To determine whether the increase in apparent molecular size was due to glycosylation, a peptide-*N*-glycosidase F (PNGase F) treatment to remove *N*-linked glycans was performed on purified proteins. Following this treatment, the electrophoretic mobility of the proteins was closer to that for the predicted molecular size (Fig. 2C, lanes 2, 3, 5, and 6), indicating that deglycosylation had occurred. Immunofluorescence analysis of the two constructs showed similar patterns of reticular staining in the cytoplasm and an absence of staining in the nucleus (Fig. 2D).

### Seroreactivity of HPgV-2 RNA-positive samples with recombinant antigens.

The HPgV-2 RNA-positive samples (2) were tested for antibody reactivity to the purified recombinant proteins via slot blotting (Table 1; also see Fig. S1 in the supplemental material). Eleven of 12 HPgV-2 RNA-positive samples showed reactivity to NS4AB alone, E2 alone, or a combination of the proteins (Table 1). Consistent with previous findings using peptides, sample ABT0239N.US did not show reactivity to either recombinant HPgV-2 protein. Similarly, samples ABT0030P.US and ABT0041P.US, obtained from individuals coinfected with HPgV-2, HCV, and HIV, were nonreactive with peptides (2), although weak reactivity was noted with the recombinant proteins. Five of the 12 HPgV-2 RNA-positive samples, all of which were also reactive for HPgV-2 E2, showed reactivity to HPgV-1 E2.

**TABLE 1:** Slot blot reactivity of HPgV-2 RNA-positive samples with recombinant HPgV-2 NS4AB, HPgV-2 E2, or HPgV-1 E2

Sample	Reactivity		
	HPgV-2 NS4AB	HPgV-2 E2	HPgV-1 E2
ABT0096P.US	++	+	+
ABT0070P.US	++	++	+
ABT0188P.US	++	++	++
ABT0055A.US	++	++	+
ABT0029A.US	++	+	−
ABT0128A.US	++	+	−
ABT0239.AN.US	−	−	−
ABT0030P.US	−	+	−
ABT0041P.US	+	+	−
ABT0116A.US	++	++	++
ABT0118A.US	++	+	−
ABT0130A.US	++	++	−

### Seroprevalence among HPgV-2-viremic samples.

Recombinant HPgV-2 proteins (NS4AB and E2) or recombinant HPgV-1 E2 was coated onto magnetic microparticles and adapted for use in the Architect system, using acridinium-labeled mouse anti-human IgG for antibody detection. Evaluation of the high-throughput assay was performed with a panel of 14 HPgV-2 RNA-positive samples. Antibodies were detected in 13/14 samples (Table 2), with all 13 samples being reactive for E2 and 8 being reactive for both recombinant proteins. For the present study, we defined sensitivity as the ability to detect antibodies in the known HPgV-2 RNA-positive samples; therefore, the sensitivity (positive predictive value for detecting HPgV-2 RNA-positive samples) using HPgV-2 E2 was 92.86%, and that using NS4AB was 57.14%. ABT0239AN.US, which was originally identified as an HCV preseroconversion sample (e.g., HCV RNA positive but antibody negative), tested negative for both HPgV-2 recombinant proteins. Three samples (ABT0030P.US, ABT0035P.US, and ABT0041P.US) from two individuals that previously tested negative with the peptides and weakly positive by slot blotting were HPgV-2 E2 positive. The two bleeds for ABT0035P.US and ABT0041P.US, spaced 7 weeks apart, showed an increased viral load and a higher signal/cutoff (S/CO) value for E2 in the later bleed. With respect to HPgV-1 reactivity, 4/14 samples were HPgV-1 RNA positive, 8/14 were HPgV-1 E2 antibody positive, and 2/14 were negative for both RNA and antibodies. Reactivity for HPgV-1 markers was mutually exclusive; no samples were dually positive for both RNA and antibodies.

**TABLE 2:** Antibody responses of HPgV-2-viremic samples to recombinant antigens

HPgV-2 isolate	Antibody/RNA reactivity			Antibody reactivity to peptides^*a*^	S/CO^*b*^ for antibody reactivity to recombinant protein		HPgV-2 viral load (log RNA copies/ml)
	HCV	HPgV-1	HIV		HPgV-2 NS4AB	HPgV-2 E2	
UC0125.US^*c*^	+/+	^*c*^/+	−/−	+	9.0 (+)	4.6 (+)	6.2
ABT0096P.US	+/+	+/−	−/−	+	7.46 (+)	26.25 (+)	3.5
ABT0070P.US	+/+	+/−	−/−	*+*	12.68 (+)	23.60 (+)	5.2
ABT0188P.US	+/+	+/−	−/−	+	1.08 (+)	43.40 (+)	2.5
ABT0055A.US	+/+	+/−	−/−	+	0.23	16.04 (+)	3.8
ABT0029A.US	+/+	−/+	−/−	+	1.33 (+)	1.74 (+)	4.6
ABT0128A.US	+/+	+/−	−/−	+	0.61	2.15 (+)	4.5
ABT0239.AN.US	−/+	−/−	−/−	−	0.05	0.14	5.8
ABT0030P.US	+/+	+/−	+/+	−	0.14	4.24 (+)	6.6
ABT0035P.US^*d*^	+/+	−/+	+/+	−	0.33	8.46 (+)	5.9
ABT0041P.US^*d*^	+/+	−/+	+/+	−	0.62	11.42 (+)	6.2
ABT0116A.US	+/+	+/−	−/−	+	5.45 (+)	30.14(+)	5.2
ABT0118A.US	+/+	−/−	−/−	+	7.06 (+)	10.97 (+)	6.2
ABT0130A.US	+/−	+/−	−/−	+	12.04 (+)	16.03 (+)	3.2

a Peptide reactivity testing was performed as described previously (2).

b The cutoff value was based on the population median plus 7 SDs.

c Limited volume was available for testing of the index case, UC0125.US; the sample was diluted 1:1 in normal human serum that had been prescreened for HPgV-1 and HPgV-2 RNA and antibodies. The sample was not screened for antibodies to HPgV-1 E2 due to insufficient testing volume.

d Samples were obtained from the same donor, with bleed dates 7 weeks apart.

### HPgV-1 and HPgV-2 seroprevalence studies.

**FIG 3:**
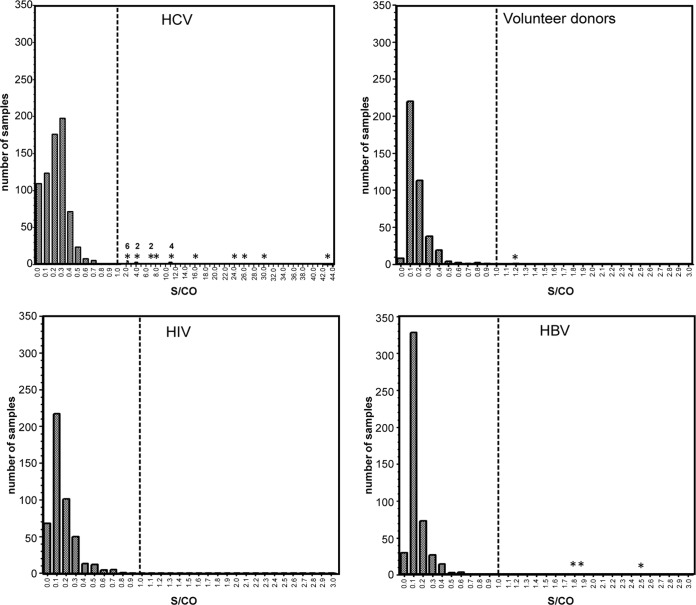
Distribution of S/CO values for anti-HPgV-2 E2 reactivity. Numbers of samples are plotted on the *y* axes, and the corresponding S/CO values are plotted on the *x* axes. Dashed vertical lines, cutoff values of the population-based median plus 7 SDs; values greater than 1 were considered positive. *, positive samples (S/CO of >1); accompanying numbers indicate the numbers of samples. The *x* axis for the HCV data set was modified to show samples with greater S/CO values.

Given the strong correlation of viremia with anti-HPgV-2 E2 responses (Table 2), this assay was selected as the primary test for expanded screening. The anti-NS4AB assay was utilized as a supplemental test to confirm reactivity among samples that were reactive for HPgV-2 peptides or E2 (Table 3). In parallel, samples were screened for antibodies to HPgV-1 E2 (Table 4). The cutoff value for the assays was set as the median signal from a HPgV-2-negative population plus 7 SDs, requiring samples to have a substantial signal to be identified as reactive (Table 4 and Fig. 3). Populations that had been tested previously for antibodies to HPgV-2 peptides and for HPgV-2 and HPgV-1 RNA were evaluated for antibodies to recombinant HPgV-2 E2. In total, 31/2,331 samples were repeatedly reactive for HPgV-2 E2, with 22/31 showing supplemental evidence of HPgV-2 exposure (Table 3). If we consider the remaining 9 samples to be nonconfirmed and potentially false-positive samples, then the specificity was 99.61%. Consistent with previous findings (2), the greatest prevalence of antibodies to HPgV-2 E2 was found among HCV-infected donors (3.31%). Of the 24 E2 antibody-positive samples, 18 samples had supporting evidence for exposure to HPgV-2 (Table 3). Among the HCV antibody-positive/HCV RNA-negative samples, 3 (1.00%) of 299 had supporting evidence of exposure. The seroprevalence among non-HCV groups was 0.30% (4/1,348 samples), with 2 samples having supporting evidence of HPgV-2 exposure. None of the samples from volunteer donors, HIV-infected subjects, or HBV-infected subjects was HPgV-2 RNA positive, including the 4 seroreactive samples.

**TABLE 3:** Prevalence of antibodies to HPgV-2 in HCV-infected, HIV-infected, HBV-infected, and volunteer control samples

Reference group	No. HPgV-2 antibody positive	Antibody prevalence (% [95% CI])	*P* value^*a*^	No. (%) positive by supplemental testing/total no.^*b*^				
				RNA	NS4AB (RNA)	Peptide (RNA)	NS4AB and peptide (RNA)	NS4AB, peptide, or RNA
HCV infected^*c*^ (*n* = 726)	24	3.31 (2.13–4.88)	NA	11	11 (7)	14 (7)	11 (7)	18/24 (75.00)
HCV infected (RNA negative/antibody positive) (*n* = 299)	3	1.00 (0.21–2.90)	0.0502	1	1 (1)	2 (1)	1 (1)	2/3 (66.67)
HIV (*n* = 434)	0	0.00 (0.00–0.85)	0.0001	0	0 (0)	0 (0)	0 (0)	0/0 (0.00)
HBV (*n* = 456)	3	0.66 0.14–1.91	0.0023	0	2 (0)	0 (0)	0 (0)	2/3 (66.67)
Volunteer donors (*n* = 416)	1	0.24 (<0.01–1.33)	0.0002	0	0 (0)	1 (0)	0 (0)	0/0 (0.00)
Total non-HCV (*n* = 1,306)	4	0.31 (0.08–0.78)	0.0001	0	2 (0)	1 (0)	0 (0)	2/4 (50.00)
Total (*n* = 2,331)	31	1.33 (0.91–1.88)	NA	12	14 (8)	17 (7)	12 (8)	22/31 (70.97)

a *P* values were calculated using Fisher's exact test, comparing the HCV group with the HIV, HBV, and volunteer control groups. NA, not applicable.

b Supplemental testing was performed for HPgV-2 RNA, anti-NS4AB, or peptide reactivity.

c The sample set includes only HCV RNA-positive/antibody-positive samples; it does not include the previously identified samples UC0125.US and ABT0239AN.US (HCV RNA positive only). The longitudinal bleed samples ABT0035P.US and ABT0041P.US were counted as 2 samples in the analysis.

**TABLE 4:** Comparison of HPgV-1 and HPgV-2 prevalence rates using molecular and serological tools

Group	Total no. (%) positive (RNA or antibody)		*P* value^*a*^	No. (%) RNA positive		No. (%) RNA and anti-E2 positive/total no. anti-E2 positive		No. (%) RNA and anti-E2 positive/total no. RNA positive		*P* value^*a*^
	HPgV-1	HPgV-2		HPgV-1	HPgV-2	HPgV-1	HPgV-2	HPgV-1	HPgV-2	
HCV infected^*b*^ (*n* = 726)	354 (48.76)	24 (3.31)	0.0001	67 (9.23)	11 (1.51)	3/287 (1.04)	11/24 (45.89)	3/67 (4.47)	11/11 (100.00)	0.0001
HCV antibody positive/RNA negative (*n* = 299)	173 (57.86)	3 (1.00)	0.0001	15 (5.02)	1 (0.33)	1/158 (0.63)	1/3 (33.33)	1/15 (7.86)	1/1 (100.00)	0.1250
HIV infected (*n* = 434)	181 (41.71)	0 (0.00)	0.0001	51 (11.75)	0 (0.00)	2/130 (1.54)	0/0 (0.00)	2/51 (3.92)	0/0 (0.00)	NA
HBV infected (*n* = 456)	53 (11.62)	3 (0.66)	0.0001	8 (1.75)	0 (0.00)	2/45 (4.44)	0/3 (0.00)	2/8 (25.00)	0/0 (0.00)	NA
Volunteer control (*n* = 416)	55 (13.22)	1 (0.24)	0.0001	17 (4.09)	0 (0.00)	0/38 (0.00)	0/1 (0.00)	0/17 (0.00)	0/0 (0.00)	NA
Total (*n* = (2,331)	816 (35.00)	31 (1.33)	0.0001	158 (6.78)	12 (0.51)	8/658 (1.21)	12/31 (38.71)	8/158 (5.06)	12/12 (100.00)	0.0001

a *P* values were determined using Fisher's exact test, comparing HPgV-1 and HPgV-2. NA, not applicable.

b The group does not include the previously identified samples UC0125.US and ABT0239AN.US (HCV RNA positive only). Overall, of the 14 known HPgV-2 RNA-positive samples, 13 (92.86%) had detectable anti-E2 responses.

The total prevalence (sum of reactivity for RNA and anti-E2 reactivity) for HPgV-1 was significantly higher (*P* > 0.0001) than that for HPgV-2 in all populations tested (Table 4). While HPgV-2 RNA was detected only in HCV-infected individuals, HPgV-1 RNA was detected in all populations tested (Table 4). Similarly, the prevalence of antibodies to HPgV-1 E2 was significantly higher (*P* > 0.0001) in all populations tested, whereas antibodies to HPgV-2 E2 were most frequently detected among HCV-positive individuals (Table 4). For HPgV-2, 12/31 anti-E2-reactive samples (38.71%) were RNA positive; in contrast, 8/664 HPgV-1 anti-E2-reactive samples (1.21%) were RNA positive, thus supporting earlier reports that the HPgV-1 E2 antibody response is a marker of resolved infections (17). With respect to the coassociation of RNA and antibodies, all 12 HPgV-2 RNA-positive samples (100.00%) were anti-E2 positive, whereas only 8/158 HPgV-1 RNA-positive samples (6.78%) were anti-E2 positive.

### Testing for HPgV-2 antibodies in HCV and HBV seroconversion panels.

In an effort to identify a longitudinal sample displaying HPgV-2 seroconversion, 68 HBV and 42 HCV seroconversion panels were tested for HPgV-2 antibodies. The first and last bleeds (when available) were tested for antibodies to E2 or NS4AB; however, no seroconversion panels yielded positive results (see Fig. S3 in the supplemental material). Due to the high predictive value of anti-E2 for HPgV-2 RNA positivity, only selected panel members were tested for HPgV-2 RNA, and none had detectable HPgV-2 RNA.

### Concordance between peptide- and recombinant protein-based antibody tests.

Twenty-nine of the 36 peptide antibody-positive samples from the original study were available for antibody testing using the recombinant proteins (2). In total, 16 of the 29 samples reactive for peptides were reactive for one or both recombinant proteins. The concordance between antibody detection using recombinant proteins and that using peptides was high for P16 (NS4AB; 93.75%) and P9 (NS5A; 56.25%) but low for P4 (NS3; 6.25%). Nine of the 13 discordant results were originally identified as P4 reactive. The 14 HPgV-2 RNA-positive samples were analyzed for correlations between individual peptide reactivity and the presence of HPgV-2 RNA (Table 5). The positive predictive values of peptide reactivity for the presence of HPgV-2 RNA were greatest for P16 (64.28%) and P9 (50.00%) but much lower for P4 (21.43%) (Table 5). Thus, antibody detection with P4 had the lowest positive predictive value for RNA and the weakest concordance with antibody detection using recombinant proteins. With the elimination of samples reactive only for P4, the results for 16/20 peptide-positive samples (80.00%) were concordant with results obtained using recombinant proteins.

**TABLE 5:** Correlation between HPgV-2 peptide and recombinant protein results

Peptide	No. (%) of samples positive for recombinant antigen and peptide/total no.	No. (%) of HPgV-2 RNA-positive samples with peptide reactivity/total no.^*a*^
P4 (NS3)	1/16 (6.25)	3/14 (21.43)
P9 (NS5A)	9/16 (56.25)	7/14 (50.00)
P16 (NS4AB)	15/16 (93.75)	9/14 (64.28)

a Reactivity was based on an S/CO value of >1, from the study by Berg et al. (2).

## DISCUSSION

Here we developed antibody tests for detection of HPgV-2 employing two recombinant proteins (NS4AB and E2), and we used the tests to obtain updated seroprevalence estimates of HPgV-2 infections. More-sensitive recombinant antibody tests detected 13 (92.86%) of 14 HPgV-2-viremic samples from 13 individuals, compared to only 10 (71.43%) of 14 samples using tests based on synthetic peptides (Table 2). Notably, antibodies were detected in 13 of 14 samples using the recombinant E2 protein, while only 8 of 14 samples were detected using the recombinant NS4AB protein. Using the more reliable E2 antibody test as the primary screen, these results strengthened the previous observation (2) of higher HPgV-2 seroprevalence among HCV-infected donors (24/726 subjects [3.31%]), compared to non-HCV-infected donors (4/1,348 subjects [0.30%]). Selection of recombinant proteins with low levels of identity to other known viruses, establishment of a conservative cutoff value for determining antibody reactivity, and the use of supplemental markers validated the seroprevalence data results.

The HPgV-2 envelope protein E2 used for antibody detection was expressed in mammalian cells to allow for glycosylation and was purified under native conditions, to maintain more closely the conformation of the envelope protein. The use of E2 resulted in the detection of antibodies in two serially collected HPgV-2 RNA-positive samples (ABT00035P.US and ABT00041P.US), collected 7 weeks apart, that had previously tested negative for antibodies to peptides and NS4AB (2). The observation of greater viral load and increased antibody reactivity in the later bleed is consistent with active replication in the presence of E2 antibodies (Table 2), similar to findings observed in chronic HCV infections.

Stringent measures were taken to ensure that the antibody tests utilizing the HPgV-2 and NS4AB recombinant proteins were specific. First, the amino acid identity of the chosen protein targets for HPgV-2 and either HCV or HPgV-1 was low, approximately 20% (see Fig. S2B in the supplemental material), thus reducing the risk of cross-reactivity. Second, a conservative cutoff value was established, requiring reactive samples to have robust signals, compared to the majority of samples. Lastly, 22/31 anti-E2-reactive samples (70.97%) had supporting evidence of HPgV-2 infection, using RNA, detection of antibodies to NS4AB, or peptide reactivity. Among 31 anti-E2-positive samples, 12 were HPgV-2 RNA positive (8 were also anti-NS4AB positive), and 10 were antibody reactive for either NS4AB or one or more peptides. The remaining 9 anti-E2-positive samples may represent resolved infections, with antibodies persisting after viral clearance, or false-positive results. In the case of HCV infections, residual reactivity for one marker is not uncommon after viral clearance, and such samples could be classified as nonconfirmed (22). It is difficult at present to define the true specificity of the anti-E2 assay, as this is a newly discovered virus and not many tools are available for comparison. If all 9 samples were false-positive samples, then the specificity of the anti-E2 test would be 99.61%. Discovery of longitudinal samples infected with HPgV-2 is critical for characterization of the antibody response with respect to the serological markers that may be present during different periods of infection (i.e., viremia and IgM and IgG responses).

To date, HPgV-2 viremia has been strongly associated with HCV coinfection (1, 2). In this study, the prevalence of anti-E2 was higher in the HCV-infected population than in the non-HCV-infected population. Among 31 anti-E2-reactive samples, 27 were associated with active or past HCV infections, while 4 samples were not associated with HCV (3 HBV-infected subjects and 1 volunteer donor). Supplemental testing using anti-NS4AB indicated resolved HPgV-2 infections for 2 of the 4 samples, suggesting that infection by this virus may not be exclusively associated with HCV. The higher prevalence of HPgV-2 among HCV-infected individuals may be related to the mode of transmission, as the vast majority of HCV infections are contracted through parenteral exposure, while the primary mode of transmission is not confined to parenteral exposure in other populations (HBV- or HIV-infected donors) (23, 24). It is also conceivable that HCV coinfection could be conducive to establishing or maintaining concurrent HPgV-2 viremia. Although the association of HPgV-2 RNA with active HCV replication was strong, we identified one sample (ABT0130A.US) as being HPgV-2 RNA and antibody positive (Table 2) but associated with a resolved HCV infection (RNA negative and antibody positive). This observation is consistent with the findings of Kapoor et al., in which 1 of 4 HPgV-2 RNA-positive samples was negative for HCV RNA (1). Notably, the samples used in our study were from apparently healthy blood donors with newly diagnosed HCV, HIV, or HBV infections, rather than subjects with symptomatic chronic disease, among whom the prevalence of HPgV-2 may be higher.

For most flaviviruses (West Nile virus, dengue virus, yellow fever virus, and HPgV-1), the resolution of infection is associated with the presence of antibodies to an envelope glycoprotein (8, 10–13). While 75% of HPgV-1-infected individuals develop antibodies to E2 and subsequently clear the virus from the blood (14, 17), the remaining 25% develop chronic infections characterized by persistent viremia. However, HCV establishes chronic infections in 75% of infected individuals despite the continuing persistence of antibodies to E2 (25). Similar to HCV and in contrast to HPgV-1, antibodies to HPgV-2 E2 are frequently detected in viremic samples (Table 4) and do not appear to indicate viral clearance in all cases.

The serological analysis presented here reveals the stark differences in the prevalence of HPgV-1 and HPgV-2 in donor populations. The total prevalence rates (detection of RNA and/or E2 antibodies) among the populations tested (*n* = 2,331) were 35.00% and 1.33% for HPgV-1 and HPgV-2, respectively (Table 4). The prevalence rates for HPgV-1 (48.76%) and HPgV-2 (3.31%) among actively HCV-infected individuals were higher than those observed among volunteer donors or those infected with HIV or HBV (Table 4). HPgV-1 RNA was detected in all groups tested (6.78%), whereas HPgV-2 RNA was restricted to the HCV-infected group (1.51%). Similarly, the presumably nonpathogenic and transfusion-transmitted anelloviruses have been detected in all of these groups, including healthy donors, and have distributions comparable to that of HPgV-1 (26–28). Thus, there is a notable dichotomy in which commensal viruses (anelloviruses and HPgV-1) are more prevalent across all populations, with HPgV-2 showing clear enrichment in populations coinfected with the pathogenic virus HCV. Although evidence of any clinical association with HPgV-2 is currently not known, the specific association with pathogenic HCV may be relevant to HPgV-2 infections.

With these new tools, we now possess the ability to detect both active and resolved HPgV-2 infections. In screening populations that share blood-to-blood contact as a route of transmission (HCV, HIV, and HBV), we have noted active infections only in the HCV-infected group, but the low antibody prevalence in all groups tested suggests that HPgV-2 infections are not restricted to HCV-infected individuals. It is thus expected that over time, as larger populations are screened, we may find additional HPgV-2 cases without HCV coinfection. In particular, screening for HPgV-2 in populations at risk of parenteral transmission but without HCV coinfection (e.g., patients who have received multiple transfusions and intravenous drug users who test negative for HCV) will be of interest in finding HPgV-2 cases. As more strains of HPgV-2 from different geographic regions are revealed, molecular and serological assays will likely be refined to detect diversity and to provide a complete epidemiological picture. Continued efforts to identify more cases of HPgV-2 infection are under way, for better understanding of the clinical relevance, epidemiology, and pathogenesis of HPgV-2.

## Supplementary Material

Supplemental material
