# Supplementary material for: Antibodies to the Novel Human Pegivirus 2 Are Associated with Active and Resolved Infections
Source: J Clin Microbiol. 2016 Jul 25;54(8):2023–30. doi: 10.1128/JCM.00515-16 (PMC4963515; doi:10.1128/JCM.00515-16)
Supplement: Supplemental material [file supp_54_8_2023__index.html]

Supplemental material 

# Antibodies to the Novel Human Pegivirus 2 Are Associated with Active and Resolved Infections

## Supplemental material

- Supplemental file 1 -

  Fig. S1 (Slot blot of HPgV-2 RNA-positive samples), S2 (Alignment of the HPgV-2 peptide 16 sequence with the corresponding region in HPgV-1 and HCV antigen 5-1-1 and alignment of HPgV-2, HPgV-1, and HCV E2), and S3 (S/CO values for testing HCV and HBV seroconversion panels for anti-E2)

  PDF, 134K
