# Supplementary material for: Antibodies to the Novel Human Pegivirus 2 Are Associated with Active and Resolved Infections
Source: J Clin Microbiol. 2016 Jul 25;54(8):2023–30. doi: 10.1128/JCM.00515-16 (PMC4963515; doi:10.1128/JCM.00515-16)
Supplement: Supplemental material [file JCM.00515-16_zjm999095081so1.pdf]

Supplemental Figures:

Supplemental Figure 1: Slot blot of HPgV-2 RNA positive samples. HPgV-2 RNA positive samples were tested for reactivity to NS4AB, HPgV-2 E2, or HPgV-1 E2 by slot blot.

Supplemental Figure 2: A. Alignment of HPgV-2 peptide 16 sequence with corresponding region in HPgV-1 and HCV antigen 5-1-1. B. Alignment of HPgV-2, HPgV-1, and HCV E2.

Supplemental Figure 3: S/CO values for testing HCV and HBV seroconversion panels for anti-E2. The following HCV and HBV seroconversion panels were tested: [panel number (bleed number)]: HCV: 100071 (1, 7), 10043 (1, 10), 913 (1, 4), 914 (1, 9), 919 (1, 7), 921 (1, 11), 922 (1, 6), 6224 (1, 6) 6229 (1, 8), 9044 (1, 6), 9047 (1, 10), 9054 (1, 10), 9058 (1, 5), 10025 (1, 11), 10041 (1, 3), 10165 (1, 9), 9055 (1, 11), 10017 (1, 16), 10002 (1, 7), 10004 (1, 6), 10008 (1, 10), 10011 (1, 13), 10020 (1, 13), 10023 (1, 21), 10029 (1, 16), 1051 (1, 14), 9054 (1, 8), 10016 (1, 12), last bleed only: 6222, 6227, 9041, 6216, 6213, 6225, 6228, 10026, 10062, 10021, 10003, 915, 6226, 10000. HBV: 6272 (1, 26), 26982/14399 (1, 25), 11007 (1, 14) 43527/3453 (1, 26), PHM935A (1, 20), 11009 (1, 23), 1808 (1, 21), 11028 (1, 10), 1002 (1, 6), 6282 (1, 14), 11031 (1, 16), 11012 (1, 6), 9279 (1, 7), 11005 (1, 14), 6271 (5), PHM925 (1, 5), 6284 (1, 19), 6274 (1, 7), 6273 (1, 6), 11017 (1, 14), 11000(1, 9), PHM (928 (1, 7), PHM912 (1, 9), PHM924 (1, 5), PHM908 (1, 8), PHM918 (1, 3), PHM904 (1, 3), PHM933(2, 6), PHM906(1, 5), PHM934 (1,6), 11014 (1(12), 11027 (1, 14), 6281 (1, 12), 6278 (1, 9), 6290 (1, 12), 11008 (1, 18), 11059 (1, 9), 11013 (1, 35), 11029 (1, 13), 11024 (1, 14), 11062 (1, 12), 11016 (1, 10), 11052 (1, 13), 11069 (1), 11006 (17), 6277 (1, 11), 6275 (1, 7), 6286 (1, 9), 1672/3471 (1, 9), 13867/3482 (1, 31), 11056 (1, 11), PHM917 (1, 3), 1807/3463 (1, 25), 26022/14518 (1,2), 9074 (1, 20), PHM927 (1, 6), PHM926 (1, 8), PHM929 (1,9), 11001 (1,8), 0994/3457 (1,23), 11026 (1,16), PHM935B (21, 32), last bleed only: 11011, 6278, 11003, 6285, PHM930, 11056. Seroconversion panels tested for HPgV-2 RNA: 6222, 6224, 6225, 6226, 6227, 6228, 9041, 9045, 9047, and 9054.

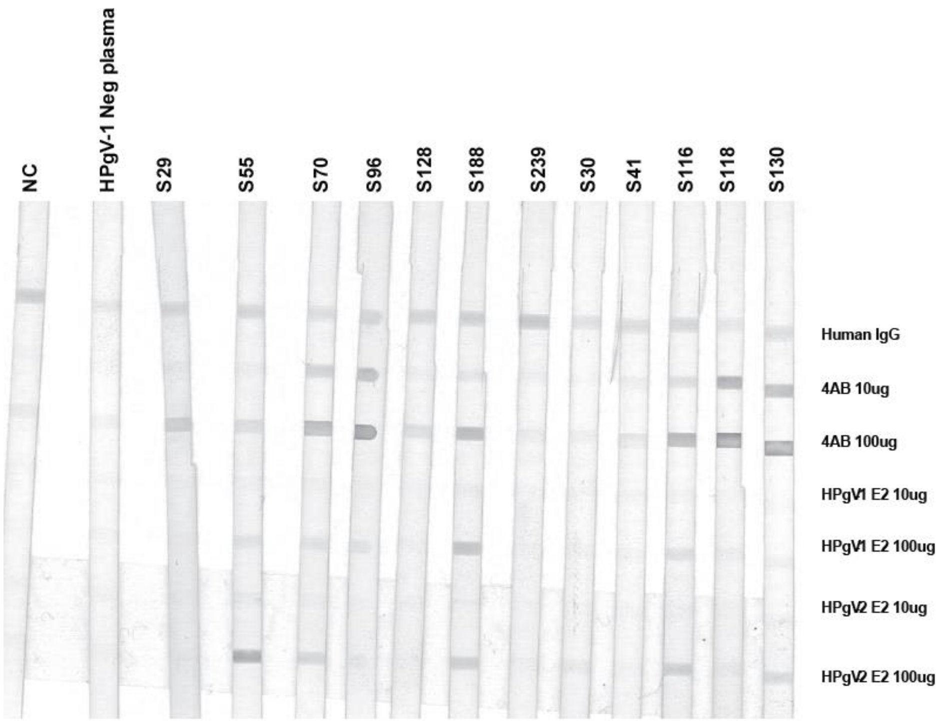

Supplemental Figure 1: Slot blot of HPgV-2 RNA positive samples. HPgV-2 RNA positive samples were tested for reactivity to NS4AB, HPgV-2 E2, or HPgV-1 E2 by slot blot.

```
A                                   1850      1860      1870      1880      1890      1900      1910      1920
                           ---------+---------+---------+---------+---------+---------+---------+---------+-----
HPgV-2                     ------SVEVR-------------------------PAGVTRPDATDETAAYAQRLYQACADSGIFASLQGTASAALGKLADASR   1889
HPgV-1                     VVVTDWDVKGGGNPLYRNGDQATPQPVVQVPPVDHRPGGESAPSDAKTVTDAVAAIQVNCDWSVMTLSIGEVLALAQAKTAEAYT   1630
5-1-1 (HCV)                ------------------------------------------IIPDREVLYREFDEMEECSQHLPYIEQGMMLAEQFKQKALGLL     43
```

```
B                                10        20        30        40        50        60        70        80
                        ---------+---------+---------+---------+---------+---------+---------+---------+
HPgV-2 E2                YKHQSESYLKYCTITNTSTSMNCDCPFGTFTRNTESRFSIPRFCPVKINSSTFICSWGSWWWFAENITRPYT------DV          74
HPgV-1 E2                --GAPASVLGSRPFEAGLTWQSCSCRSNGSRVPTGERVWERGNVTLLCDCPNGPWVWVPALCQAIGWGDPITHW----SH          74
HCV E2                   --HVTGGNAG--RTTAGLVGLLTPGAKQNIQLINTNGSWHINSTALNCNESLN-TGWLAGLFYQHKFNSSGCPERLASCR          75

                                 90       100       110       120       130       140       150       160
                        ---------+---------+---------+---------+---------+---------+---------+---------+
HPgV-2 E2                GMPPAPISALCYIYSNN------DPPPWYHNT----------TIIPQNCRNSTVDPTTAPCRD------KWGN--ATACI         130
HPgV-1 E2                GQNQWPLSCPQFVYGAVSVTCVWGSVSWFASTGGRDSKVDVWSLVPVGSASCTIAALGSSDRDTVVELSEWGIP-CATCI         153
HCV E2                   RLTDFAQGWGPISYANGSGLDE-RPYCWHYPPRPCGIVPAKSVCGPVYCFTPSPVVVGTTDRSG-APTYSWGANDTDVFV         153

                                170       180       190       200       210       220       230       240
                        ---------+---------+---------+---------+---------+---------+---------+---------+
HPgV-2 E2                LDRRSRFCGDCYGGCFYTNGSHDRSWDR--CGIG--YRDGLIEFVQLGQIRPNISNTTIEL---LAGASLVIASGLRPGF         203
HPgV-1 E2                LDRRPASCGTCVRDCWPETGSVRFPFHR--CGAGPRLTRDLEAVPFVNRTTPFTIRGPLGN---QGRGNPVRSPLGFGSY         228
HCV E2                   LNNTRPPLGNWFGCTWMNSTGFTKVCGAPPCVIGGVGNNTLLCPTDCFRKHPEATYSRCGSGPWITPRCMVDYPYRLWHY         233

                                250       260       270       280       290       300       310       320
                        ---------+---------+---------+---------+---------+---------+---------+---------+
HPgV-2 E2                GCSRAHGVVHCYRCPSYRDLEQFGPGLGKWVPLPGEPVPELCINPQWARRGFRMSNNPLSLLQTFVEDIFLAPFCNPTPG         283
HPgV-1 E2                TMTKIRDSLHLVKCPTP----AIEPPTGTFGFFPGVPPLNNCMLLGTEVSEVLGGAG---LTGGFYEPLVRR--CSELMG         299
HCV E2                   PCTINYTIFKVRMYVGG-----VEHRLEAACNWTRGERCDLEDRDRSELSPLLLSTTQWQVLPCSFTTLPALS-TGLIHL         307

                                330       340       350
                        ---------+---------+---------+-------
HPgV-2 E2                RVRVCNNTAFYPRGGGFVQLIGDVQVLTPNT                                                  314
HPgV-1 E2                R-R-------NPVCPGFAWLS                                                            312
HCV E2                   HQNIVDVQYLYGVGSSIASWAIKWEYVVLLFLLLADA                                            344
```

Supplemental Figure 2

Supplemental Figure 2: A. Alignment of HPgV-2 peptide 16 sequence with corresponding region in HPgV-1 and HCV antigen 5-1-1. Peptide 16 sequence is highlighted. B. Alignment of HPgV-2, HPgV-1, and HCV E2.

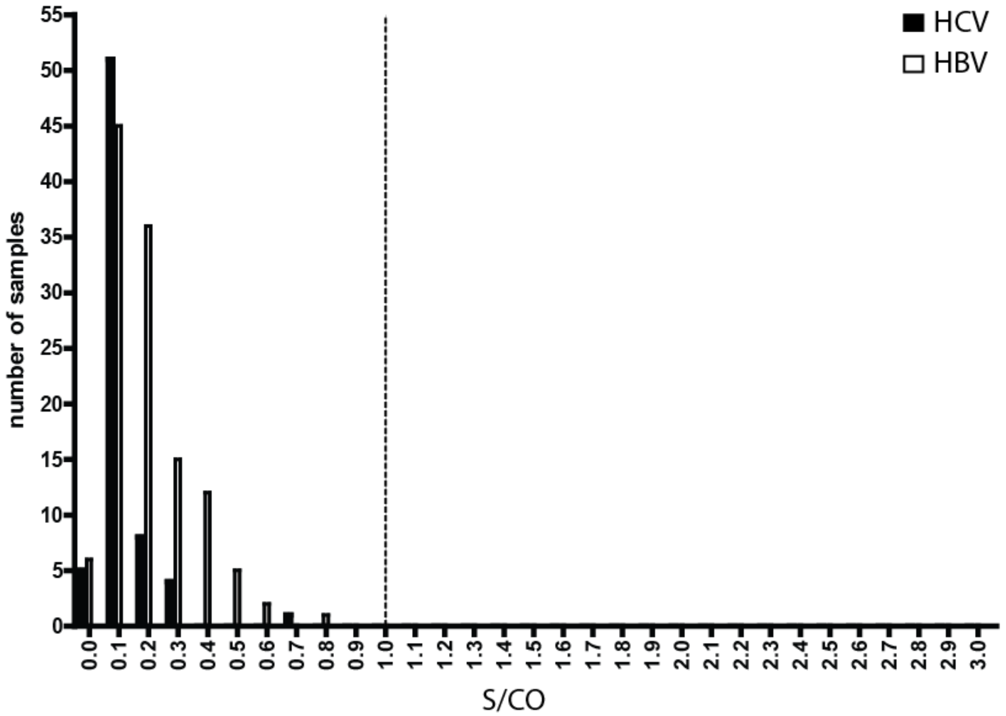

Supplemental Figure 3: S/CO values for testing HCV and HBV seroconversion panels for anti-E2. The following HCV and HBV seroconversion panels were tested: [panel number (bleed number)]: HCV: 100071 (1, 7), 10043 (1, 10), 913 (1, 4), 914 (1, 9), 919 (1, 7), 921 (1, 11), 922 (1, 6), 6224 (1, 6) 6229 (1, 8), 9044 (1, 6), 9047 (1, 10), 9054 (1, 10), 9058 (1, 5), 10025 (1, 11), 10041 (1, 3), 10165 (1, 9), 9055 (1, 11), 10017 (1, 16), 10002 (1, 7), 10004 (1, 6), 10008 (1, 10), 10011 (1, 13), 10020 (1, 13), 10023 (1, 21), 10029 (1, 16), 1051 (1, 14), 9054 (1, 8), 10016 (1, 12), last bleed only: 6222, 6227, 9041, 6216, 6213, 6225, 6228, 10026, 10062, 10021, 10003, 915, 6226, 10000. HBV: 6272 (1, 26), 26982/14399 (1, 25), 11007 (1, 14) 43527/3453 (1, 26), PHM935A (1, 20), 11009 (1, 23), 1808 (1, 21), 11028 (1, 10), 1002 (1, 6), 6282 (1, 14), 11031 (1, 16), 11012 (1, 6), 9279 (1, 7), 11005 (1, 14), 6271 (5), PHM925 (1, 5), 6284 (1, 19), 6274 (1, 7), 6273 (1, 6), 11017 (1, 14), 11000(1, 9), PHM (928 (1, 7), PHM912 (1, 9), PHM924 (1, 5), PHM908 (1, 8), PHM918 (1, 3), PHM904 (1, 3), PHM933(2, 6), PHM906(1, 5), PHM934 (1,6), 11014 (1(12), 11027 (1, 14), 6281 (1, 12), 6278 (1, 9), 6290 (1, 12), 11008 (1, 18), 11059 (1, 9), 11013 (1, 35), 11029 (1, 13), 11024 (1, 14), 11062 (1, 12), 11016 (1, 10), 11052 (1, 13), 11069 (1), 11006 (17), 6277 (1, 11), 6275 (1, 7), 6286 (1, 9), 1672/3471 (1, 9), 13867/3482 (1, 31), 11056 (1, 11), PHM917 (1, 3), 1807/3463 (1, 25), 26022/14518 (1,2), 9074 (1, 20), PHM927 (1, 6), PHM926 (1, 8), PHM929 (1,9), 11001 (1,8), 0994/3457 (1,23), 11026 (1,16), PHM935B (21, 32), last bleed only: 11011, 6278, 11003, 6285, PHM930, 11056. Seroconversion panels tested for HPgV-2 RNA: 6222, 6224, 6225, 6226, 6227, 6228, 9041, 9045, 9047, and 9054.
